# Blood–brain barrier alterations and their impact on Parkinson’s disease pathogenesis and therapy

**DOI:** 10.1186/s40035-024-00430-z

**Published:** 2024-07-29

**Authors:** Kristina Lau, Rebecca Kotzur, Franziska Richter

**Affiliations:** 1 Department of Pharmacology, Toxicology and Pharmacy, University of Veterinary Medicine Hannover, Foundation, Bünteweg 17, 30559 Hannover, Germany; 2 Center for Systems Neuroscience, Hannover, Germany

**Keywords:** Neurovascular unit, Synucleinopathies, α-Synuclein

## Abstract

There is increasing evidence for blood–brain barrier (BBB) alterations in Parkinson’s disease (PD), the second most common neurodegenerative disorder with rapidly rising prevalence. Altered tight junction and transporter protein levels, accumulation of α-synuclein and increase in inflammatory processes lead to extravasation of blood molecules and vessel degeneration. This could result in a self-perpetuating pathophysiology of inflammation and BBB alteration, which contribute to neurodegeneration. Toxin exposure or α-synuclein over-expression in animal models has been shown to initiate similar pathologies, providing a platform to study underlying mechanisms and therapeutic interventions. Here we provide a comprehensive review of the current knowledge on BBB alterations in PD patients and how rodent models that replicate some of these changes can be used to study disease mechanisms. Specific challenges in assessing the BBB in patients and in healthy controls are discussed. Finally, a potential role of BBB alterations in disease pathogenesis and possible implications for therapy are explored. The interference of BBB alterations with current and novel therapeutic strategies requires more attention. Brain region-specific BBB alterations could also open up novel opportunities to target specifically vulnerable neuronal subpopulations.

## Introduction

The blood–brain barrier (BBB) separates the delicate neuronal environment from the peripheral blood. Impairments regarding this barrier are strongly associated with inflammation and neurodegenerative diseases [1–3]. Parkinson’s disease (PD) is the second most common and currently the fastest growing neurodegenerative disease, predicted to reach 12.9 million cases by 2040 with highest prevalence in aging society [4]. Loss of dopaminergic neurons in the substantia nigra pars compacta and consequent lack of dopamine lead to the hallmark motor-symptoms of PD: bradykinesia, tremor and postural instability [5]. While these symptoms still define PD as a movement disorder, it is increasingly recognized that pathologies in other brain regions and even in peripheral organs significantly contribute to the clinical picture and the disease burden [6]. Hence, PD is not restricted to the dopaminergic system. Instead, PD can be referred to as a multi-system syndrome with each patient harboring unique patterns of pathology and set of symptoms [7]. This becomes especially apparent in non-motor symptoms like sleep disturbances, anxiety, depression, cognitive deficits and gastrointestinal impairments [8]. While the pathogenesis of PD is still elusive, abnormal cytoplasmic protein deposits termed Lewy bodies, can be found throughout the brain and the body of PD patients [7]. The distribution of Lewy bodies is not random but seems to progress in a schematic way. It initially appears in the olfactory bulb and dorsal motor nucleus of the nervus vagus located in the lower brain stem and progresses to the substantia nigra and striatum [7, 9]. An important protein aggregated inside of Lewy bodies is α-synuclein; therefore PD is classified as a synucleinopathy [10]. Mutations in or multiplications of the gene for α-synuclein (*SNCA*) are causative for rare familial forms of PD. The majority of PD cases are idiopathic with likely multifaceted pathogenesis [11]. Detrimental processes of α-synuclein misfolding together with mitochondrial dysfunction, neuroinflammation and lysosomal deficits, among others, are currently accepted mechanisms in idiopathic PD [12–14]. α-Synuclein aggregates exhibit prion-like characteristics and when insoluble α-synuclein fibrils are injected into the brains of animals, this pathology can template soluble α-synuclein and thereby spread to other brain regions [15, 16]. To this day, there is no cure or neuroprotective treatment for PD and although dopamine replacement alleviates some symptoms, its use is limited by development of dyskinesias. Most non-motor symptoms remain untreatable and severely reduce the quality of life for patients [5, 17]. Of note, current symptomatic therapy options and future disease-modifying treatment strategies could be interfering with BBB alterations and associated inflammatory reactions in PD [18]. Here we provide an overview of BBB alterations observed in and associated with PD. Additionally, we highlight possible pathways of PD pathogenesis connected to BBB alterations and how this could influence therapeutic strategies.

## Anatomy and physiology of BBB

### BBB anatomy: structure and composition

Blood vessels infiltrating the brain and the spinal cord form the BBB [19]. The cerebral and spinal capillaries are covered by endothelial cells connected to each other by tight junction proteins, forming the inner part of the BBB [19]. Tight junction proteins provide a tightness necessary for physiological barrier function. They prevent paracellular diffusion of ions and small molecules and thereby contribute to electrical, ionic and molecular homeostasis [20]. The neuronal signal needs to be reproducible, precise and reliable, therefore the neuronal tissue has to be separated from any ion fluctuations that occur in the blood due to food intake or peripheral neurotransmitter release [20]. Other junctional molecules present at the BBB include adherens junctions and gap junctions. While adherens junctions like cadherins connect to the cytoskeleton [21, 22], gap junctions form channels between neighboring endothelial cells, consisting of two connexons, each containing six transmembrane proteins called connexins [23]. Adherens junctions are therefore important regulators of trans-endothelial migration and modulate receptor signaling [24–26], whereas gap junctions enable intercellular communication [23].

Towards the abluminal side, the endothelial cells are wrapped by a thin basement membrane, mostly consisting of extracellular matrix protein collagen IV [3]. The basement membrane does not only cover endothelial cells, but also surrounds pericytes. Some studies reported that pericytes are able to regulate blood flow due to their ability to contract [27–30]. Moreover, they support the integrity of the BBB through direct contact and paracrine signaling [30, 31].

Astrocytes enwrap the capillary with their end-feet, providing a cellular link between neuronal brain tissue and blood vessels [3]. They stimulate contraction/relaxation of pericytes and thereby regulate the blood flow according to neuronal activity. This so-called neurovascular-coupling is mediated by local glutamate levels [3, 32, 33].

The BBB, together with astrocytes, pericytes, capillary-associated microglia (CAMs), perivascular macrophages and the immediate surrounding neuronal tissue, forms the neurovascular unit [34]. The neurovascular unit compromises highly active endothelial and glial cells adjacent to neurons [35]. This unit allows neurovascular coupling with feed-forward and feed-back mechanisms regulating nutrient and oxygen supply to neuronal tissues in a brain region-specific manner [34]. While parenchymal microglia connect to the vasculature via their processes, CAMs use their more stable somata to interact with the capillary [36]. The interaction of microglia with the vessels seems to be dual: processes of microglia can efficiently restore BBB integrity but once they turn reactive, the secreted cytokines may increase invasion by immune cells [37, 38]. Perivascular macrophages are embedded in the basement membrane and have an important role in immune regulation [39]. Additionally, they are able to take up and scavenge intracerebroventricularly injected fluorescently labeled macromolecules [40, 41]. Although the role of neurovascular unit in immune surveillance and the concepts of neurovascular interaction remain to be fully clarified, it is believed that dysfunction in neurovascular coupling could affect the BBB and the neuronal tissue [35, 42].

### BBB physiology: barrier functions

#### Endothelial glycocalyx

The endothelial cells express a luminal network consisting of proteoglycans, glycoproteins and soluble molecules [43], which is called the endothelial glycocalyx. The endothelial glycocalyx prevents solutes to reach the endothelium not only by providing a physical barrier, but also due to its negative charges [44–46]. Negatively charged molecules show a significantly lower crossing rate in in-vitro experiments than neutral ones [47], while cationization of molecules increases their crossing abilities [48, 49]. Therefore, the endothelial glycocalyx contributes to the impermeability of the barrier. Not only molecules are restricted to reach the endothelium, also cell-vessel interaction is influenced by the endothelial glycocalyx through limiting the contact of red blood cells and platelets with the endothelium and attenuating the adhesion of leukocytes to cell adhesion molecules like intercellular adhesion molecule-1 (ICAM-1) and vascular adhesion molecule-1 (VCAM-1) [43, 50, 51].

#### Pericytes

Pericytes are contractile cells covered by the basement membrane that maintain BBB integrity [29, 30]. Via their contractility they can regulate blood flow and oxygen supply according to neuronal demand. Acute pericyte loss results in aberrant hemodynamic responses due to a lack of capillary dilation in response to neuronal activity [52]. Moreover, widespread pericyte loss over time leads to hypoxia in brain tissues and metabolic stress, which result in abnormal neuronal excitability and neurodegeneration [53, 54]. This could be validated by several recent studies which implicate that pericyte dysfunction is an early sign for cognitive decline [55, 56]. Pericytes are also able to respond to and initiate an inflammatory response [57]. For instance, during acute phase of stroke, pericytes first participate in pro-inflammatory response causing BBB disruption, and they also provide neuroprotection by stabilizing the BBB in a later disease stage [58].

#### Astrocytes

Astrocytes provide a bridge between neuronal tissue and blood vessels, which is termed neurovascular coupling [32, 33]. They do not only regulate contraction of pericytes in response to neuronal activity, but are also able to induce the barrier-like phenotype of endothelial cells [32]. In cultured endothelial cells, astrocytes stimulate the formation of tight junctions through secretion of various factors (e.g., basic fibroblast growth factor-β and angiopoietin) and thereby tighten the barrier [59]. Several glial tumors lacking these inductive factors exhibit a leakier BBB than the surrounding brain tissue [3, 60]. Besides regulating blood flow to meet the neuronal energy demand, the neurovascular coupling potentially facilitates physiological ‘tightening’ of the barrier during hypoxic stress or physiological ‘opening’ of the barrier to allow increased glucose supply or enhanced lymphocyte migration [60–62].

Water-channels (aquaporins) are membrane proteins on astrocytic end-feet, located specifically at the area where astrocytic end-feet connect to capillaries [60]. The localization of aquaporins correlates with the expression of agrin on the basement membrane, a heparin sulfate proteoglycan that anchors aquaporin in close proximity to the blood vessel [60]. Aquaporins are important for regulating water homeostasis and for edema prevention [63, 64]. Aquaporin-4 is the key regulator of water flow and K^+^ uptake in the central nervous system (CNS) [65, 66]. To this purpose, aquaporin-4 mediates the distribution of K^+^ throughout the brain according to the local K^+^ concentration [67]. High K^+^ levels around astrocytic end-feet increase their local K^+^ uptake and induce K^+^ efflux in distant cell processes [67]. Interestingly, aquaporin-4 is associated with neurodegenerative diseases and its level is decreased in Alzheimer`s and PD patients [68–70].

### BBB physiology: tightly controlled transport

#### Free diffusion and active efflux

The BBB is a highly sealed endothelium layer securely connected by tight junction proteins [20]. However, enabling free diffusion to some extent is necessary for brain metabolism and pH regulation. Specialized endothelial cells forming the BBB lack intracellular fenestration and therefore the molecular size threshold for free diffusion across the BBB is considered to be 400–600 Da [71], 72]. However, Pan et al. injected CINC1 (a chemokine of 7.8 kDa) intravenously in mice and found no saturation of CINC1 crossing at the BBB, indicating a diffusion-limit at a higher molecular mass than originally stated [73]. It is important to note that not only the size, but also the level of lipophilicity can determine the effectiveness of free diffusion at the BBB [74]. In addition, many lipophilic molecules are substrates of multidrug efflux pumps, which limit the rate of netto-uptake by the brain [75]. Therefore, it could appear as if molecules do not diffuse through the layer, while in fact they are actually quickly removed even via mechanisms not yet discovered for the given molecule [76].

The most prominent multidrug efflux pump is P-glycoprotein (P-gp), a synonym for multidrug resistance protein-1 [77]. P-gp is a member of the ATP-binding cassette (ABC) transporter family, which are ATP-driven efflux pumps [75]. Localized at the luminal side of the endothelial cells, P-gp is able to immediately remove diffused substrates back into the blood [76, 78, 79]. Their broad substrate range, including drugs and xenobiotics, and their high expression levels at the BBB, contribute a major part to CNS pharmacoresistance [75]. Several mechanisms are involved in the regulation of P-gp expression [80]. Rapid reactions include posttranslational modifications and activation by P-gp activators [80, 81]. A delayed but long-lasting response to chronically increased levels of neurotoxic compounds in blood circulation includes transcriptional mechanisms and posttranscriptional modifications regulating P-gp expression [80, 81]. To date, several microRNAs are known to upregulate P-gp expression (including miR-138 and miR-296), while others downregulate P-gp expression (including miR-1253 and miR-298) [82]. P-gp can be transferred through cell–cell contacts, in extracellular vesicles and intracellularly [80].

#### Carrier-mediated transport across BBB

A selective blood-to-brain influx/efflux system allows for a more restricted transport across the BBB. Nutrients and other supplements are required to reach the neuronal tissue at a high rate, which is facilitated by endothelial solute carrier-mediated transport [83]. Glucose, which is the key energy metabolite in the CNS, is transported into the brain via a concentration gradient by carbohydrate transporters, most prominently, glucose transporter-1 (GLUT1) [84, 85]. GLUT1 is located on both the luminal and the abluminal sides [86]. Another energy metabolite, lactate, is transported across the BBB by monocarboxylate transporter-1 (MCT1; MCT2 in rodents), which is expressed equally on luminal and abluminal sides of endothelial cells [86, 87]. Hormones and all essential amino acids have substrate-specific transporters, although cationic amino acids (lysine and arginine) or large neutral ones (tryptophan, tyrosine) share a transporter system (cationic amino acid transporters 1 and 3 (CAT-1/3); *L*-type amino acid transporters 1 and 2 (LAT-1/2)) [88–91]. Both, CAT-1/3 and LAT-1/2 are expressed on both sides of endothelial cells [92]. The ubiquitously expressed LAT-1 transport system transports *L*-dopa across the BBB [93]. A disadvantage of amino acid transporters is the competition between therapeutic compounds and amino acids. While in primates *L*-dopa uptake by the brain is reduced after a high protein meal [94], amino acid supplementation in PD patients treated with *L*-dopa has no effect on neurological parameters [95]. Moreover, essential fatty acids, nucleotides, amines, cholines, organic anions and cations, as well as vitamins, are being transferred into the brain through carrier-mediated transport [83].

#### Receptor-mediated transport across BBB

Receptor-mediated transport provides another possibility for some molecules such as transferrin and low-density lipoprotein to pass the BBB [96]. Although receptor-mediated transport and carrier-mediated transport are 10–100 times faster than free diffusion, they are saturable transport systems [97]. This could be a strategy to avoid accumulation of neuroactive peptides in the brain [98]. For example, transferrin, the major source of iron in the brain, is transported across the BBB via transferrin receptor (TfR) [99]. TfR is localized at the luminal and the abluminal sides of capillaries and serves as a bidirectional transporter [100, 101]. The increased iron levels in the substantia nigra lead to the hypothesis that iron metabolism disorders and iron-mediated oxidative stress may be involved in neurodegeneration [102–104]. Ferritin is an iron storage molecule that can bind several Fe^3+^ molecules, thereby contributing to iron homeostasis [105]. By binding to TfR, both transferrin and ferritin can reach the brain [106].

Low-density lipoprotein receptor related protein-1 (LRP-1) and receptor for advanced glycation end products (RAGE) are important members of the receptor-mediated transport system. They not only carry the substrates included in their names, apolipoproteins and glycosylated proteins, but also amyloid-beta [107, 108]. The luminally expressed RAGE acts as an influx transporter for amyloid-beta [108], while the abluminally expressed LRP-1 initiates amyloid-beta clearance via transcytosis in brain-to-blood direction [109]. LRP-1 knockout mice with endothelial-specific LRP-1 deficiency exhibit BBB leakage [110, 111]. LRP-1 ablation leads to transcriptional activation of the self-autonomous pathway CypA-MMP9, resulting in increased levels of cyclophilin A (CypA) and matrix-metalloprotease (MMP)-9 as detected by real-time PCR and immunohistochemistry [111]. Subsequent degradation of tight junction proteins and neurodegenerative processes due to BBB impairment could be prevented by a non-immunosuppressive CypA inhibitor, Debio-25 [111]. This emphasizes that LRP-1 is not only an important transporter system, but also a crucial regulator of BBB integrity.

#### Cellular trafficking across the BBB

Apart from substances like drugs, toxins or ions, peripheral immune cells may invade the brain through the BBB, thereby representing an important bridge between the peripheral and the central immune response. Immune cells crossing the BBB require several steps that include rolling (mediated mostly via selectin), activation, arrest, crawling and transmigration [112]. After activation of surface adhesion molecules, leukocytes bind to endothelial receptors like ICAM-1 and VCAM-1 [112]. Expression of ICAM-1 and VCAM-1, as well as selectin, is strongly linked to inflammation. However, even under healthy conditions, some lymphocytes may enter the brain to participate in immune surveillance [113, 114].

## Structural and functional changes of the BBB in PD

### Evidence for increased BBB permeability in human postmortem tissues

Early attempts to study BBB permeability in PD did not detect overt disruptions, although the presence of mononuclear cells infiltrating the substantia nigra and the striatum suggested neuroinflammation, a major BBB disruptor [115–117]. In more recent and detailed studies of post-mortem fixed striatum tissues of PD patients, extravasation of serum proteins like albumin and fibrinogen and red blood cells were detected [118–120]. Other groups detected CD8^+^ and CD4^+^ T-cells in the substantia nigra pars compacta in postmortem human brain tissues [121]. It is important to note that the loss of BBB integrity could occur in the aging brain. However, Montagne et al. found BBB breakdown associated with physiological aging only in hippocampal CA1 and dentate gyrus regions, while other brain regions remained unaffected [55]. Obviously, a major limitation of any study conducted with postmortem tissues is the inability to differ between initial insults and subsequent alterations. In order to gain such insights, albumin leakage into the cerebrospinal fluid of healthy controls and age-matched patients at early and late disease stages was measured. Disease progression was classified by Unified PD Rating Scale part III (early disease stage: 15–25; late disease stage: 30–50) and Hoehn-Yahr staging (early disease stage: 1–2; late disease stage: 2.5–4). While early-stage patients had similar levels of albumin as controls, advanced patients showed a significant increase of albumin levels in their cerebrospinal fluid [120]. Although albumin was only measured in the cerebrospinal fluid, it marks impairments of the blood-cerebrospinal fluid barrier and the BBB. Increased levels of albumin in the cerebrospinal fluid after stroke, which injures only the BBB and not the choroid plexus, indicate that the BBB contributes to the composition and volume of the cerebrospinal fluid [120, 122, 123]. However, reduced cerebrospinal fluid turnover rate and flow rate as described in patients with Alzheimer's disease (AD), could result in higher albumin levels in the cerebrospinal fluid of patients in later disease stage [124, 125]. If BBB integrity is impaired during disease onset or progression, neuronal tissue will be exposed to detrimental substances circulating in the blood and thereby may accelerate progression of the disease, as further discussed in the Section “Evidence for a role of BBB alterations in disease pathogenesis”.

**Fig. 1:**
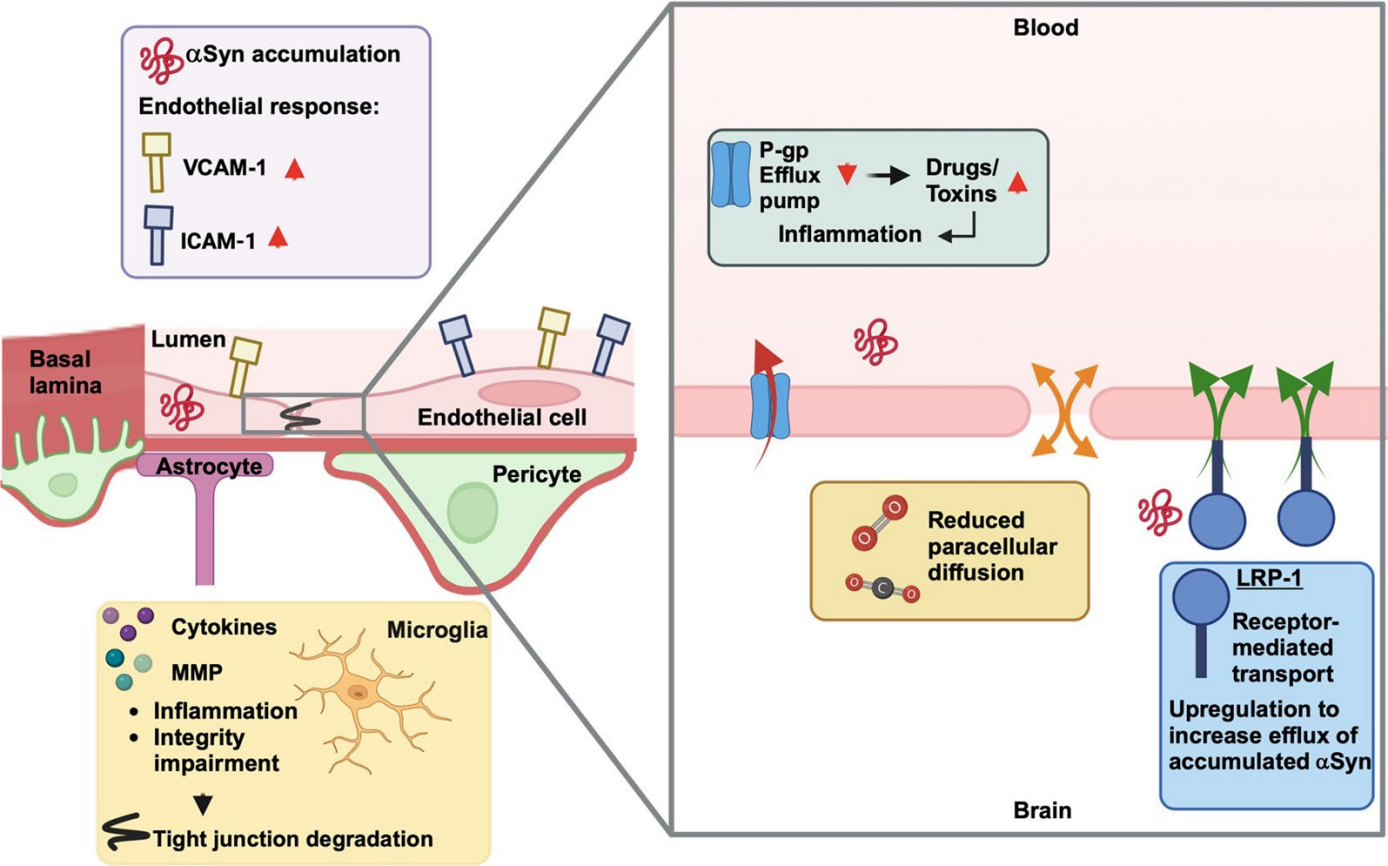
Proposed impact of blood–brain barrier alterations on Parkinson’s disease. Endothelial cells, linked by tight junction proteins, form the inner part of the BBB. The basement membrane, which encircles the endothelial cells, is shared with pericytes, which enwrap the endothelial cell tube. Astrocytic end-feet connecting to the capillary, as well as adjacent microglia and brain parenchyma, represent the abluminal side of the barrier. Small lipophilic molecules such as O_2_ and CO_2_ can cross the endothelial cell layer via free diffusion. Other molecules are held back and rely on specific transporters. Intracellular accumulation of aggregated proteins like α-syunclein could trigger an inflammatory response. Upregulation of cell adhesion molecules like VCAM-1 and ICAM-1 by activated endothelial cells could leave the BBB more prone to peripheral immune cell invasion as they enable leukocytes to migrate through the endothelial cell layer. Cleavage of α-synuclein by MMPs results in neurotoxic formations, contributing to the on-going and evolving inflammatory status. Cytokines released by activated pericytes and endothelial cells stimulate microglia, which will subsequently impair the integrity of the barrier. By degrading tight junction proteins, MMPs have a similar impact on the BBB. P-gp is an efflux pump with a broad substrate range, pumping (toxic) substances out of the brain. A reduced function of P-gp may lead to an increase in toxic substances, further increasing the inflammatory stimuli. LRP-1 is a more specific protein that induces a receptor-mediated transport. LRP-1 seems to transport amyloid-beta and α-synuclein in an efflux manner. Increased levels of LRP-1 might be an attempt to adjust to increase protein loads. ICAM-1: intercellular adhesion molecule-1; VCAM-1: vascular adhesion molecule-1; P-gp: P-glycoprotein; LRP-1: low-density lipoprotein receptor-related protein 1; MMP: matrix-metalloprotease; αSyn: α-synuclein

In line with increased permeability, altered expression of tight junction proteins has been found in postmortem brain tissues from PD patients [126] (Fig. 1). In particular, occludin and zonula occludens-1 seem to be affected by α-synuclein pathology in the brain [126]. The same effects on zonula occludens-1 and occludin have been found on hCMEC/D3 cells incubated with α-synuclein PFFs [126].

### α-Synuclein and the BBB: dysregulation of major transporters

Immunohistochemistry of the wall of leptomeningeal vessel in human brain tissues demonstrated intense staining of α-synuclein in endothelial and smooth muscle cells [127]. Interestingly, intraparenchymal vessel stained only weakly for α-synuclein, and the wall of cerebral capillaries was negative for α-synuclein [127]. The mechanisms regulating α-synuclein expression and its overall function in endothelial cells are still unknown. Physiologically, α-synuclein is an abundant protein in brain tissues, and is present in cerebrospinal fluid and blood as well [128–130]. Thus, it may not be surprising that α-synuclein can be transported through endothelial cells across the BBB via several known mechanisms. Depending on the direction of the transport, LRP-1-mediated transport, Clathrin-mediated transcytosis followed by an early endosome–retromer–late endosome trafficking pathway, and transport via extracellular vesicles have been described [131–133].

LRP-1 is a receptor associated with α-synuclein transport in the abluminal-to-luminal direction. LRP-1 is found upregulated in postmortem PD brain tissues [131, 134] (Fig. 1). In aging rodents, LRP-1 expression is decreased [109]. Moreover, post-mortem studies of brains from AD patients indicate reduced expression of LRP-1 and increased RAGE (influx pump) expression, leading to a net increase of amyloid-beta influx into the brain [135]. Sui et al. demonstrated that α-synuclein is capable of inhibiting amyloid-beta efflux, suggesting that LRP-1 might be involved in α-synuclein efflux as well [131]. The observed upregulation of LRP1 could be a clearance mechanism in response to the α-synuclein pathology.

RAGE acts as an influx pump for amyloid-beta and is increased in patients with AD [108, 135]. Recent data indicate that RAGE also interacts with α-synuclein [136]. Moreover, RAGE levels are increased in the cortex of PD patients [137] (Fig. 1). The binding of α-synuclein to RAGE significantly increases the pro-inflammatory response [136]. When a substrate binds to the receptor, a cascade is initialized that leads to expression of pro-inflammatory molecules like NF-κB [138]. However, the impact of RAGE on the pathology of PD by stimulation of inflammatory responses, possibly through binding to α-synuclein, is only a hypothesis that needs further studies [139, 140].

Although P-gp is not directly associated with α-synuclein transport, its function has been studied extensively in PD patients. Brain uptake of [C]-verapamil, a substrate of P-gp that is physiologically extruded quickly, was measured by positron emission tomography (PET). Brain levels of [C]-verapamil are significantly reduced in advanced PD brains [141, 142], while increased in early-stage patients [142] (Fig. 1). Several nuclear receptors are suggested to regulate P-gp expression via binding to DNA [143]. Ligands of nuclear receptors, such as 1α,25-dihydroxyvitamin D3, cross the plasma membrane and directly activate the cytosolic nuclear vitamin D receptor [81, 144, 145]. The vitamin D receptor activation leads to transcriptional upregulation of the target gene MDR1a [144, 146]. This not only increases efflux of P-gp substrates, but also alleviates cerebral amyloid-beta accumulation in transgenic AD mice [144, 147]. Reduction of P-gp levels induced by stereotactic injection of α-synuclein preformed-fibrils (PFFs) could be reversed after treatment with 1α,25-dihydroxyvitamin D3 in-vivo [146]. Moreover, in vitro experiments show that TGF-β and retinoic acid secreted by astrocytes induce P-gp expression and activity [148, 149]. A drastic reduction of the function of this important efflux pump in an already diseased brain could facilitate the accumulation of toxic compounds, causing further damage to the tissue and promoting disease progression.

It has been proposed that astrocytes can internalize α-synuclein and are involved in its spreading via direct astrocytic cell contacts and tunneling [150–152]. This uptake of α-synuclein may also lead to astrocytic activation, similar to an inflammatory stimulus [152]. In the blood, α-synuclein is mostly located in red blood cells, but it can also be secreted directly into the blood stream within extracellular vesicles [130]. Astrocytes can take up extracellular vesicles crossing the BBB, possibly spreading α-synuclein pathology in PD patients, as further discussed in Section “Evidence for a role of BBB alterations in disease pathogenesis” [133, 153].

### Inflammation

Inflammatory responses by microglia and astroglia widely spread in brains of PD patients. Although it is not clear whether BBB disruption and α-synuclein pathology are the cause or the result of inflammation, an ongoing inflammatory state will stimulate endothelial cells to contract, leading to a porous rebuilding of the endothelial cell layer [154]. Consequently, circulating blood molecules and protein-rich exudate could reach the neuronal tissue and further enhance inflammation [154]. Activation of endothelial cells could trigger the expression of VCAM-1 and ICAM-1, promoting invasion of peripheral immune cells [155] (Fig. 1). In line with this, a study using postmortem brain tissues from PD patients and controls, as well as monkeys exposed to 1-methyl-4-phenyl-1,2,3,6-tetrahydropyridine (MPTP), found over-expression of ICAM-1 in the substantia nigra of patients with PD and in the MPTP monkeys [156]. The endothelial glycocalyx restricts cell-vessel interactions and attenuates the adhesion of leukocytes to cell adhesion molecules like ICAM-1 and VCAM-1 [43, 50, 51]; however, this function is not static. In fact, blood flow and pressure, shear stress and enzymatic shedding continuously change the glycocalyx [43]. Inducing shear stress increases hyaluron expression in in-vitro experiments, indicating that the endothelial glycocalyx reacts to shear stress as a mechanotransducer [43, 157]. In rats, enzymatic shedding of the glycocalyx is primarily induced by MMPs [158, 159]. Studying possible biomarkers for endothelial glycocalyx degradation in humans is an emerging field. However, as shedding occurs in response to acute as well as chronic inflammation, correlation to a specific disease appears challenging [160].

In addition to endothelial cells, pericytes also respond to inflammatory stimuli with a phenotype change as observed in Huntington disease and PD animal models [161, 162]. The protein levels of pericyte activation markers NG2 and GFP in mice exposed to the neurotoxin 6-hydroxydopamine (6-OHDA) were significantly increased in Western blots and immunohistochemistry [161]. In neurological disorders, pericyte alterations are often associated with neuronal degeneration [162, 163]. This seems plausible, considering their functions in regulating blood flow, angiogenesis and clearance of toxins [30]. Moreover, pericytes are able to release pro-inflammatory molecules, also in response to α-synuclein exposure [57, 164–166]. Pro-inflammatory cytokines like interleukin (IL)-6, IL-1β and tumor necrosis factor-alpha (TNF-α) can cause degradation of tight junction proteins and the endothelial glycocalyx through activated MMPs and trigger a reactive phenotype of microglia [167–169] (Fig. 1). Interestingly, MMP-3 co-localizes with Lewy bodies and cleaves α-synuclein, and an upregulation of its expression could be beneficial [170]. However, it is proposed that cleavage of α-synuclein by MMP-3 results in α-synuclein fragments with higher neurotoxicity and aggregation rate [171]. This is supported by the co-localization of MMP-3 with pyroglutamate79-modified α-synuclein variant which is reported to form neurotoxic oligomers itself [171, 172]. Reactive microglia can have converse effects on the BBB. While initially trying to maintain the BBB integrity, they turn into BBB-disrupting phenotypes with persistent inflammation [37].

Interestingly, de Rus Jacquet et al. reported that astrocytes isolated from human female donors carrying the PD-associated *LRRK2* G2019S mutation are pro-inflammatory [173]. By expressing IL-6, astrocytes contribute to neuronal cell death, which can be prevented by blocking the IL-6 receptor (IL-6R) in vitro using the anti-IL-6R antibody, Tocilizumab [174]. Moreover, the isolated PD astrocytes did not support the formation of a functional BBB in an in-vitro BBB model using induced pluripotent stem cells (iPSCs) and microfluid technologies [173]. It remains unclear whether astrocytes initiate inflammatory BBB damage and if with ongoing PD pathology, they lose their ability to maintain and stabilize the barrier.

### Vessel degeneration or angiogenesis

Importantly, there are studies indicating endothelial cell degeneration, as well as an increase in string vessel formations in brains of PD patients [119]. String vessels display as empty and collapsed basal membrane tubes lacking endothelial cell and have no function in blood circulation [119, 175]. In line with this, a previous postmortem study reported decreased vessel length and branching points as well as increased vessel diameter in PD patients [176].

Interestingly, in contrast to these findings, angiogenesis is hypothesized to be present in brains of PD patients, induced by stressors like hypoxia or inflammation [177, 178]. Angiogenesis includes the formation of new blood vessels in the adult brain and is thus not only restricted to embryonic formation of blood vessels [179]. In postmortem tissues of PD patients the number of endothelial cells seems to be 2.5-fold higher than that in healthy control brains [180]. Moreover, integrin αvβ3-positive vessels and other angiogenic markers are present in the brains of PD patients [177, 181, 182]. Integrin αvβ3 is an adhesion molecule restricted to angiogenic vessels [183]. It is important to note that neither new vessel formation nor an increase in vessel density was detected [177]. Beneficial restoration of blood vessels to distribute nutrients and oxygen can also increase the risk of edema, due to increased number of fenestrae, wider endothelial cell junctions and discontinuous basement membrane of the newly formed vessels [177, 184]. These dual effects are a major risk factor regarding therapeutic angiogenesis in stroke and also in AD, where hypervascularization due to extensive angiogenesis is expected to impair BBB integrity [184, 185]. Furthermore, it cannot be ruled out whether angiogenesis is secondary to inflammation or whether it promotes the inflammatory response in a self-perpetuating manner in PD [177]. This implies that angiogenesis and vascular degeneration do not exclude each other but instead could co-occur in a dynamic and stage-dependent process.

### Challenges of BBB assessments in health and diseases

It remains unclear to what extent BBB alterations precede inflammation and α-synuclein accumulation or display as a result of these and other pathologies. This is a major limitation of any study conducted with postmortem brain tissues. The BBB is not a static structure. As mentioned before, blood pressure, shear stress, inflammation and locally shifted energy demand all provoke continuous reorganization of the barrier. Therefore, changes in BBB during early disease progression could be overlooked when studying post-mortem tissues only. BBB characteristics like permeability and receptor expression could be altered specifically in one brain region and missed when studying whole brain tissues. Some regional differences in BBB structure are physiological, such as increased permeability of the microvasculature surrounding circumventricular organs like the area postrema, subfornical organ, organum vasculosum laminae terminalis, pineal gland, posterior pituitary, intermediate lobe of pituitary gland, median eminence and subcommissural organ [186]. The endothelial cells of the blood vessels surrounding the circumventricular organs express less tight junction proteins, hence exhibit a fenestrated phenotype [187]. It is suggested that astrocytes, tanycytes and the outer basement membrane function as a second barrier around these organs to prevent solutes to reach the rest of the brain [188]. Interestingly, the permeability of the barrier seems to differ also among other brain regions under physiological conditions. For example, imaging studies revealed greater permeability in the thalamus than in the putamen [189]. When assessing permeability between healthy and diseased brains, these regional differences should be taken into account. There are not only regional differences, but also differences between capillaries, arterioles and venules. A method that allows detection of cell variation between the arteriovenous axis known as “zonations” is single-cell sequencing [190]. Cell variation within one group of cells can be detected. A molecular atlas of the human brain vasculature has been constructed [190], and it further provides the possibility to identify pathologic transformation of cells and of the vascular pattern in diseased brains [191]. For example, proposed vascular degeneration in AD has been validated by sequencing nuclei extracted from isolated vessels, which revealed decreased number of vascular cell nuclei in AD patients compared to control [192]. Arterioles, in general, seem to express less P-gp and have thicker astrocytic sheath, while capillaries are surrounded by more contractive astrocytic processes [193, 194]. Out of those three, venules are described to express the highest amount of inflammation-associated proteins, and capillaries highly express solute transporter systems [195]. These differences, just like the differences among brain regions, should be considered when studying BBB alterations in tissues or isolated capillaries. Studying isolated capillaries to characterize endothelial cells could contribute to the understanding of how these cells react to the ongoing pathology. Using in-vitro systems to test endothelial cell reaction to α-synuclein pathology or inflammation, is a useful tool also considering transport of proteins across the barrier. However, these systems lack the full brain anatomy such as interfering astrocytes, microglia and neurons as well as PD pathophysiology, and are therefore limited when studying disease progression. It is important to mention that nearly all the PD patients are treated with levodopa, which itself can have major impacts on the BBB [196–198].

## BBB dysfunction in models of PD

Studying the BBB in PD mechanistically requires a suitable model of the barrier that also develops PD-relevant pathology. PD is characterized by loss of dopaminergic neurons in the substantia nigra pars compacta and the presence of aggregated, intracellular inclusions containing α-synuclein, termed Lewy bodies [199]. In vitro models of the BBB are commonly used to study the permeability of the cell layer or drug entry through the barrier [200]. BBB models consisting of more than the endothelial cell layer are superior as additional cells like astrocytes and pericytes induce tight junction expression via paracrine or juxtacrine signaling [59]. To better understand cellular mechanisms of PD pathology in affected cells, patient-specific iPSCs are available [200]. Cultivation of these cells is preferably done on a permeable membrane with either no medium flow or with medium flow through a hollow fiber on which the cells grow [200, 201]. The latter one is more physiological by providing some curvature and shear stress; nonetheless, all in-vitro models lack complete anatomical structure and interaction between all involved cell types [200]. Cultivating human dopaminergic neurons of the substantia nigra on a Brain-Chip that recreates the neurovascular unit is a recently established method to study BBB disruption due to PD pathology [202, 203]. Exposing this model to α-synuclein PFFs induces neuronal cell death, BBB permeability as well as altered expression of genes associated with autophagy, oxidative stress, mitochondrial dysfunction, and inflammation [202]. However, models of neurodegenerative diseases and the neurovascular unit on a chip are not yet widely used and reproduced [204]. To more faithfully model the BBB in PD, in-vivo models are required. Several non-mammalian, invertebrate models exist to model environmental and genetic factors of PD (e.g., the baker`s yeast *Saccharomyces cerevisiae*, the nematode *Caenorhabditis elegans*, the fruit fly *Drosophila melanogaster*) [205]. They all breed quickly in large numbers and have genetic profiles matching up to 75% of disease-related genes in humans [206–208]. Moreover, these animals allow studying of neurotransmitters, ion channels, receptors and transporters [205]. However, these animals lack similar brain structures and capillary network as their blood (hemolymph) is flowing freely in the body cavity [209]. Still, a structure similar to the mammalian BBB can be found, consisting of two surface glia cell types (apical perineurial glia and basal subperineurial glia) and extracellular matrix [210, 211]. These two glia cell types exhibit septate junctions with the same function as tight junctions in the mammalian BBB: preventing hemolymph with high levels of potassium to reach the nerves [210]. Additionally, these animals also express ABC-efflux transporters as a homolog to P-gp [211]. However, given all these differences from the human BBB, it is not surprising that BBB alterations are not frequently studied in these PD models. To combine the versatile advantages of invertebrates with similarity of mammalian anatomic structures, zebrafish are used to model PD using genetic and toxin-based approaches [212]. The BBB of zebrafish is surprisingly similar to the BBB of mice and humans. It is formed by the same structures such as basal membrane, endothelial cells, astrocytes and pericytes. Results of permeability and drug availability studies also correlate to those derived from mouse studies [213, 214]. Therefore, the model is commonly used to investigate BBB penetration of drugs rather than investigation of BBB alterations itself [215]. Furthermore, inducing PD-like phenotypes and disease mechanisms in these models is largely limited to few specific pathologies. Thus, modeling the complexity of the BBB in PD requires vertebrate models. The available rodent models are mirroring symptoms and/or pathological features of the disease, albeit no model encompasses all aspects [17]. One approach is to induce dopaminergic neurodegeneration by administering neurotoxins locally or systemically [216]. Stereotactic injection of 6-OHDA due to its inability to cross the BBB, and systemic application or stereotactic injection of MPTP, are the most frequently used approaches [217]. Both toxins cause oxidative stress and rapid neuronal loss [218], but they fail to represent progression, non-motor symptoms and protein misfolding pathomechanisms [218–220]. Moreover, administration of neurotoxins can cause direct neuroinflammation and damage to cell-populations, blurring the effects of dopaminergic cell loss. A different approach with higher construct validity is using well-known disease-causing mutations to generate animal models [216]. For example, autosomal dominant point mutations in the α-synuclein (*SNCA*) gene as well as duplication or triplication of the *SNCA* gene are causative for PD [221–225]. These models are a useful tool to study disease mechanisms as well as disease progression [219]. Non-human primates are highly similar to humans and extremely sensitive to MPTP and 6-OHDA, and mirror the clinical phenotypes of PD [226, 227]. However, experiments with non-human primates need to remain an exception due to reasonable ethical concerns.

Thus far, data on BBB alterations are largely limited to rodent models of PD. In order to precisely describe these findings related to the underlying pathology, toxin-based and genetic animal models are described separately.

### BBB alterations in toxin-based rodent models of PD

The neurotoxins 6-OHDA and MPTP have different characteristics considering BBB crossing and reaching. 6-OHDA needs to bypass the BBB and is therefore injected stereotactically into the brain [228]. MPTP and other neurotoxins like the pesticide Paraquat can penetrate the BBB due to their high lipophilicity (MPTP) or via neutral amino acid transporter (Paraquat) [229–231]. BBB permeability assessments, like Fluorescein isothiocyanate (FITC) permeability assay and detection of edema using a MRI scan, are frequently performed in toxin-based animal models and suggest that MPTP or 6-OHDA exposure leads to increased leakage [232–235]. MPTP-induced barrier dysfunction is independent from dopaminergic neurodegeneration, as TNF-α knock-out mice display neurodegeneration but no BBB leakage [236]. Consequently, the MPTP-induced barrier breakdown is likely a result of acute TNF-α-dependent neuroinflammation [236]. Moreover, the period of widespread albumin leakage is short (resolved after 12 h) while lymphocytes are able to migrate through the barrier long after, due to lasting cellular and molecular changes [121]. Upregulation of intercellular adhesion molecule-1 in capillaries of mice exposed to MPTP could enhance immune cell invasion rate [121, 156]. The 6-OHDA-induced BBB leakage persisted even 34 days after injection [232]. As microarray analysis indicated upregulation of inflammatory genes shortly after injection of 6-OHDA [237], it may be possible that BBB leakage is first triggered by neuroinflammation and then driven by dopaminergic cell loss [236]. Padel et al. hypothesized that dopamine loss induced by 6-OHDA injection could impair the angiogenic vascular response and trigger pericyte activation, as they observed an increase in pericyte number and morphological changes at the site of insult [161]. Of course, effects of the neurotoxins themselves should be taken into consideration: 6-OHDA and MPTP are highly effective neurotoxins inhibiting respiratory processes leading to formation of free radicals and oxidative stress, which could extend beyond the dopaminergic neurons [218, 229]. Reactive oxygen species are reported to influence endothelial cells and the BBB in general, inducing cytoskeletal rearrangements and increased permeability [238, 239]. The severity of the impact of 6-OHDA and MPTP is further evident in a significant reduction of tight junction proteins such as claudin-3, occludin and zonula occludens-1 [233, 234]. Whether such BBB altering effects of toxins are representative of the human disease may be questionable; however, 6-OHDA represents byproducts of toxic dopamine metabolism and MPTP represents environmental exposure to pesticides [240, 241]. Mitochondrial deficiency is observed in brain tissues of PD patients, and some familial mutations causing PD impact mitochondrial function and mitophagy [242–244]. Thus, these pathways and their potential impact on the BBB remain likely candidates among the pathogenic factors driving neuronal loss.

Western blotting and immunohistochemistry analysis detected increased levels of RAGE in striatal neurons and astrocytes, but not in microglia, in mice exposed to MPTP [245]. Also, in other animal models using chronic MPTP application, lipopolysaccharide (LPS), rotenone and 6-OHDA, RAGE expression was found only in neurons and not in microglia [246–248]. This could indicate that pro-inflammatory response is not the only function of RAGE in the pathology of PD as suggested by Gasparotto et al. [139]. Both, MPTP and 6-OHDA exposure seem to increase the expression of MMPs [233, 249]. MMPs can degrade basement membrane and tight junction proteins, like occludin and claudin-5, thereby contributing to BBB damage [250]. Chung et al. found in mice that deficiency of MMP protects nigrostriatal dopaminergic neurons from MPTP toxicity and prevents BBB damage, as well as microglial activation [235]. This further indicates that BBB impairments most likely result from neuroinflammation in toxin-based models.

Besides that, MPTP-injected primates and 6-OHDA-exposed rats also exhibit angiogenic characteristics, where β3-integrin and vascular endothelial growth factor are found to be upregulated [232, 251]. Moreover, blood vessel volume and blood vessel density in the substantia nigra are increased in primates after MPTP treatment [251]. As discussed above for PD patients, it can be argued that these changes are more a reaction to inflammation and hypoxia and less to nigrostriatal neuronal loss.

In general, it should be noted that the toxin-based models replicate nigrostriatal dopaminergic cell loss and neuroinflammation, but lack progressive characteristic and overt α-synuclein pathology [218]. Therefore, it is challenging to translate these findings to PD patients. Still, most of the BBB alterations described in toxin models mirror those found in post-mortem brain tissues, supporting the scenario that neuroinflammation impairs the BBB in PD. Lacking the progressive nature, toxin-based models fail to answer the important question, i.e., whether BBB alterations precede and/or contribute to neuroinflammation.

### BBB alterations in genetic rodent models of PD

The risk of developing PD is partly manifested in genetics either by identified disease-causing mutations or genetic risk factors for idiopathic PD [252]. Autosomal-dominant point mutations in the *SNCA* gene (A30P, A53T and E46K) have been identified and linked to familial PD [221–224]. Intending to replicate familial PD, transgenic mice expressing the mutated human α-synuclein were generated. These animals develop α-synuclein aggregation and neuronal dysfunction such as disrupted autophagy [216, 253]. However, these models do not develop overt and specific dopaminergic cell loss and corresponding motor deficits and are more frequently used to study α-synuclein pathology and early disease development [216]. Interestingly, duplications or triplications of the *SNCA* gene are also linked to PD and increased levels of wild-type α-synuclein can accelerate Lewy body pathology [225]. This and the fact that α-synuclein accumulates in the brains of sporadic PD patients are implemented in mouse models with over-expression of human wild-type α-synuclein. These models show slower disease progression compared to the mutated α-synuclein lines [254, 255]. Under the broadly expressed Thy1-promotor (Thy1-αSyn), there is widespread over-expression and aggregation of human wild-type α-synuclein, leading to motor and non-motor symptoms, and loss of dopamine at around 14 months of age [255]. Over-expression can furthermore be achieved by expression under the mouse α-synuclein promotor using the bacterial artificial chromosome (BAC) construct (BAC-αSyn) [256]. These mice exhibit some motor and non-motor dysfunctions as well as dopaminergic impairments [256].

If BBB alterations can be induced by α-synuclein pathology, animals with human α-synuclein over-expression should show these alterations. Indeed, both Thy1-αSyn and BAC-αSyn transgenic lines have a reduced vessel density [257, 258]. While the number of pericytes is constant in both lines, pericytes of BAC-αSyn mice display an activated phenotype, as indicated by immunofluorescence staining for pericyte activation marker NG2 [257, 258]. This has not been determined in Thy1-αSyn mice yet. Importantly, both α-synuclein and pS129-αSyn, which is associated with potentially toxic aggregates [259], are deposited in endothelial cells [257, 258]. In immunohistochemically stained isolated capillaries from Thy1-αSyn mice, the astrocytic end-feet marker aquaporin-4 is decreased compared to wild-type controls [257]. Yet it remains unclear whether astroglial dysfunction or aquaporine-4 dispersion is the cause [257]. Astrocytic dysfunction in response to α-synuclein accumulation has been described previously, but was mostly restricted to their activation rather than degradation [260]. Importantly, specific dispersion of aquaporine-4 could result in a structural loss of astrocytes, leading to a deficient support for the BBB [64, 261]. Additionally, aquaporine-4 is an important mediator for astroglial and microglial communication; therefore, its downregulation could hint at a dysfunction of microglia-astroglia coupling [62, 262].

**Fig. 2:**
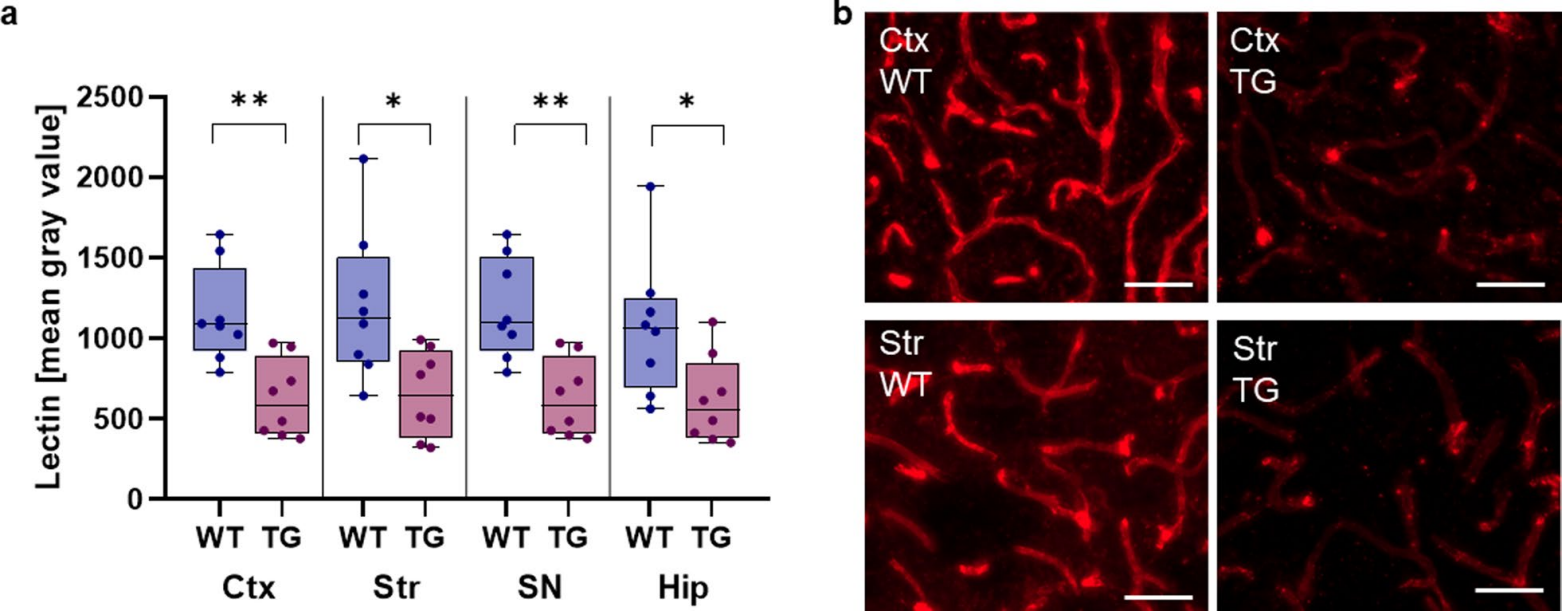
Decreased lectin intensity in Thy1-αSyn mice. **a** Measurement of lectin intensity in 4 different brain regions (Ctx: cortex, Str: striatum, SN: substantia nigra, Hip: hippocampus) of Thy1-αSyn transgenic mice (TG) compared to wild-type (WT) animals (*n* = 8 per group) at the age of 6 months. All values are presented as mean ± SEM, individual data points are shown. *P < 0.05, **P < 0.01, Mann–Whitney test. **b** Representative fluorescent images of brain slices stained by lectin (red) in 2 brain regions (Ctx: cortex, Str: striatum) of a 6-month-old Thy1-αSyn (TG) mouse and a wild-type (WT) mouse. Scale bars, 50 µm. To measure lectin intensity, brains were treated following protocols reported previously [265]. After blocking with goat serum, slices were incubated with DyLight649-conjugated Lycopersicon esculentum (tomato) lectin (1:200, Invitrogen, #L32472) overnight at 4 °C. Sections were mounted on glass slides using ProLong™ Gold Antifade Mountant with DAPI (Thermo Fisher). Image acquisition was performed using a Zeiss Axio Observer 7 microscope equipped with an Axiocam 506, Zeiss Colibri 7 LED light source and 20 × magnification lenses. Z-stack images from each brain region (*n* = 8, cortex, striatum, hippocampus, substantia nigra) were transferred to 2-dimensional images using the sum slice function from Fiji package of ImageJ. Afterwards, the sum of gray values of all pixels divided by total pixel number was measured using the mean gray value tool from Fiji package of ImageJ [268]. Brain sections were stained in parallel by one blinded person. Imaging and quantification were performed by the same person blinded to genotype, to carefully rule out variability in staining intensity and other confounding factors

Remarkably, Western blot data showed increased protein level of LRP-1 and decreased protein level of P-gp in the isolated capillaries of the striatum of Thy1-αSyn mice [257]. This could represent a clearing mechanism in response to the aggregation of α-synuclein in the surrounding brain tissue. In general, α-synuclein over-expression seems to induce a widespread inflammatory response. Not only BAC-αSyn mice show increased microglial activation, but the mutated α-synuclein A53T protein also stimulates microglia to transform into a reactive phenotype [258, 263]. The Thy1-αSyn mice have increased levels of TNF-α and microglia reactivity, both prior to dopamine loss [264]. Moreover, the endothelial cells react to the broad α-synuclein expression by upregulating expression of ICAM-1, VCAM-1, and MMP-3, as detected in isolated capillaries [257]. In brain tissues of Thy1-αSyn mice, MMP-3 co-localizes with α-synuclein [171]. Given the fact that degradation of the endothelial glycocalyx is primarily induced by MMPs, it is not surprising that lectin intensity is remarkably reduced in several brain regions in Thy1-αSyn mice compared to wild-type controls (Fig. 2a, b) [158, 159]. Together with the previously published data of decreased vascular density in the striatum of Thy1-αSyn mice, this indicates a more widespread alteration in the endothelial glycocalyx in this model [265]. Glycoproteins, as part of the endothelial glycocalyx, have a small cytoplasmic tail, a transmembrane domain and variable extracellular domains [266]. Selectins represent a part of glycoproteins that have lectin as an extracellular domain [266]. Imaging of lectins and thereby imaging of the endothelial glycocalyx can be achieved by most plant-based fluorescently labeled lectins [43, 267]. Measuring the intensity of lectin staining to quantify the endothelial glycocalyx is a common method, and all brain sections should be stained in parallel and quantified by a person blinded to genotype, to exclude confounding factors [267].

Interestingly, BAC-αSyn mice show increased leakage of fibrinogen especially in striatal regions [258]. Expression of A53T mutated α-synuclein in astrocytes leads to BBB disruption in mice and a decrease in glutamate transport in affected astrocytes [269]. In reaction to A53T mutated α-synuclein pathology in the brain, levels of tight junction proteins zonula occludens-1, occludin and claudin-5 are reduced and FITC-dextran leakage is observed [270]. While brain region-specific alterations in tight junction expression were identified, no IgG-permeability was found in Thy1-αSyn mice [257]. However, a single dose of the bacterial endotoxin LPS leads to a measurable leakage of IgG into brain parenchyma [257]. While an impaired BBB might be more sensitive to insults, it is also possible that an increase in LPS-receptors, such as toll-like receptors, seen in animals with α-synuclein over-expression, enhances the toxicity of LPS to the BBB [264, 271]. This would imply that once α-synuclein pathology alters the BBB, the brain tissue would become more prone to bacterial endotoxins, further enhancing inflammation.

In addition, over-expression of α-synuclein can be induced by viral vectors or the administration of α-synuclein PFFs. Different from genetically modified animals, these methods can target specific brain regions and the dosage can be adjusted and administered at adult age, not interfering with early development [216, 272]. Treatment with PFFs leads to a reduction in tight junction protein expression in cell cultures while the transepithelial-transendothelial electrical resistance values are not affected [126, 202]. Six months after stereotactic injection of human α-synuclein PFFs, vessel lengths and diameter were reduced and string vessels formed [273]. The intracellular domain of LRP-1 (LRP-1-ICD) was found upregulated in capillaries from mice 6 months after PFF injection [273]. Over-expression of LRP-1-ICD via an adeno-associated virus with an endothelial-specific promotor worsened the PFF-induced motor and cognitive impairments as well as vascular damage [273]. Excessive cleavage of mature LRP-1 by the enzyme Furin could be the cause of increased LRP-1-ICD levels [273, 274]. Inhibiting Furin seems to have beneficial effects against neurodegenerative symptoms induced by cerebral ischemia [274].

In summary, α-synuclein pathology in animal models induces BBB alterations which mirror those found in patients. It seems that neuroinflammation secondary to the overt α-synuclein pathology causes BBB alterations, considering the widespread microglial activation in Thy1-αSyn, BAC-αSyn and A53T mice [258, 263, 264]. Microglia can have detrimental effects on the BBB once they turn to a phagocytic state [37]. Contrary, BBB alterations increase neuroinflammation by leaving the brain tissue vulnerable to substances in the blood. This could be a vicious cycle of neuroinflammation destabilizing the BBB, and BBB dysfunction increases neuroinflammation. Whether the α-synuclein-induced inflammation disrupts the BBB requires more research. Inhibiting inflammation in an α-synuclein-overexpression mouse model and comparing BBB alterations could be an option.

### Comparison of patient BBB alterations across all rodent models of PD

As previously described, BBB alterations found in post-mortem brain tissues of PD patients can be mirrored in animal models of the disease (for summary see Table 1). Extravasation of blood cells, including erythrocytes, CD8^+^/CD4^+^ cells and lymphocytes, has been discovered in post-mortem brain tissues of PD patients and in brains of toxin-based animal models [118, 120, 121]. Fibrinogen and albumin leakage has been detected in patients’ brain tissues, genetic animal models and toxin-based models [119, 120, 232, 236, 258, 265]. Upregulation of endothelial cell-adhesion molecules like VCAM-1 and ICAM-1 and additional detection of MMPs, emphasize that in human brain tissues, as well as in genetic and toxin-based animal models, an inflammatory neuronal status is exhibited [121, 156, 170, 233, 249, 265]. A reactive phenotype change of pericytes could be observed in both types of animal models, but was not detected in human brain tissues yet [161, 265]. As toxin-based animal models do not exhibit strong α-synuclein pathology, it is plausible that α-synuclein-positive endothelial cells are only found in genetic models and in brains of PD patients [127, 218, 265]. Altered tight junction expression is found only in genetic animal models and patients, suggesting a causative role of α-synuclein pathology [126, 265]. P-gp, the most important efflux pump located at the BBB, is reduced in genetic animal models and a reduction in its functionality is also described for late-stage PD patients [132, 141, 142]. Interestingly, P-gp may be increased in early disease stages based on current patient and model data [142, 232]. Upregulation of P-gp, which is supposed to remove toxins from the brain, could be a response to the neurotoxin in toxin-based animal models. LRP-1, which is suggested to be involved in α-synuclein transport, is upregulated in genetic animal models and in PD patients, but not in toxin-based models that do not show α-synuclein accumulation [131, 134, 265]. Upregulation of LRP-1 solely in response to α-synuclein pathology may be a compensatory clearing mechanism, further supported by the neuroprotective effects of inhibition of the LRP-1-cleaving Furin. RAGE, another transporter associated with α-synuclein, is upregulated in brain tissues of patients and toxin-based animal models, but has not been studied in genetic animal models yet [136, 137, 245]. Aquaporin-4, a water-channel located at astrocytic end-feet connecting blood vessels, is reduced in brain tissues of patients and in genetic animal models [63, 69, 265]. There is a controversy on the angiogenetic characteristics as well as vessel degeneration in the brains of PD patients [119, 176, 177, 180–182]. In genetic models only vessel degeneration has been described so far [258, 265]. On the contrary, for toxin-based animal models only angiogenetic features are detected [232, 251]. This strengthens the theory that degeneration and angiogenesis can be found simultaneously, especially as inflammation leads to endothelial cell (and subsequently also vessel) degeneration but also increases angiogenetic stimuli.

**Table 1:** Comparison of observed BBB alterations in patients with PD, toxin-based animal models and genetic models

BBB alterations	PD patients	Toxin-based models		Genetic models	
		6-OHDA	MPTP	BAC-αSyn	Thy1-αSyn
Extravasation of blood cells/molecules	Albumin [120] Fibrinogen [118, 119] Red blood cells [118] CD8^+^/CD4^+^ cells [121]	Albumin [232]	Albumin [236] Lymphocytes [121]	Fibrinogen [258]	IgG leakage after LPS exposure [265]
Altered tight junction protein expression	Occludin/ zonula occludens-1 [126]				Occludin/Zonula occludens-1/claudin-5[265]
α-Synuclein present in endothelial cells	Present [127]			Present [258]	Present [265]
LRP-1	Upregulated [134]				Upregulated [265]
P-gp	Reduced functionality [141, 142] Upregulated in early disease stage [142]	Upregulated [232]			Reduced [265]
RAGE	Increased [137]		Increased [245]		
Activation of endothelial cells	ICAM-1 upregulated [156]		ICAM-1 upregulated [121] [156]		ICAM-1, VCAM-1 upregulated [265]
Pericytes		Phenotype change [161]		Phenotype change [258]	
AQP-4	Reduced [69]				Reduced [265]
Matrix-metalloprotease	Co-localizes with Lewy bodies [170]	Upregulated [249]	Upregulated [233]		Upregulated [265]
Vessel density	Endothelial cell degeneration [119] String vessel formation [119] Decreases in vessel length and branching points and increase in vessel diameter [176]			Reduced [258]	Reduced [265]
Angiogenesis	Present [177, 178] Increase in endothelial cell number [180] Integrin αvβ3-positive vessels [177, 181, 182]	β3-integrin [232]	VEGF Increases in blood vessel volume and blood vessel density [251]		

Altogether, although genetic and toxin-based models offer completely different approaches to model PD, they show similar alterations of the BBB. Both approaches induce inflammation, supported by pronounced microglial reactivity, which disturbs BBB integrity. The resulting BBB impairment could perpetuate disease progression by leaving the neuronal tissue more vulnerable to external factors. Additionally, increased expression of cell adhesion molecules could facilitate peripheral immune cell invasion, while altered transporter proteins could interfere with physiological removal of toxins and aggregated proteins. As PD itself is a very broad and complex disease, it is not surprising that a variety of models are needed to study its pathogenesis [216]. However, taking into account that similar BBB alterations are described across several PD models and in brain tissues of PD patients, it clearly is an integral part of the pathological picture. Whether these BBB alterations play an important role in development and/or progression of the disease is an important but insufficiently answered question which requires further research. The current state of knowledge regarding this question is summarized in the next section.

## Evidence for a role of BBB alterations in disease pathogenesis

In the previous sections we summarize evidence for BBB alterations in PD. Considering the role of the BBB in health and diseases, it can be hypothesized that this pathology acts as accelerator in PD pathology and disease progression. In this section we will discuss some relevant mechanisms.

In normal aging, BBB breakdown could only be found in the hippocampal CA1 region and the dentate gyrus, and histological alterations like thickening of the basement membrane are also restricted to specific brain regions [275]. This implies that aging itself does not ubiquitously affect BBB permeability, but may underlie some brain region-specific alterations. Moreover, aging increases the vulnerability of the BBB to disruptive substances [55, 275, 276], and age-associated conditions like hypertension [277, 278] and cerebral ischemia [279, 280] alter the BBB. Moreover, head trauma accompanied by at least minor cerebral bleeding increases the risk of PD [281–283]. Recent studies have explored brain capillary damage as a potential biomarker for cognitive dysfunction [284]. The quotient of cerebrospinal fluid/serum albumin and other markers for endothelial dysfunction like vascular endothelial growth factor (VEGF), VCAM-1 and ICAM-1 are being tested [285]. A high level of pericyte injury marker platelet-derived growth-factor receptor β in the cerebrospinal fluid correlates with future cognitive decline in mouse models of AD [56, 286]. A disrupted BBB leaves the CNS open to peripheral vascular factors and immune cells, possibly initiating a degenerative process with a self-perpetuating character [287]. Further investigations on promising biomarkers could reveal tools with great power to discriminate between possible diseases and disease stages [285, 288].

Interestingly, the BBB of the striatum is very susceptible to ischemic or osmotic stress [118]. For instance, BBB permeability increases in the striatum prior to other brain regions upon ischemic stress [289]. Several brain regions affected by Lewy body pathology innervate the striatum, such as the substantia nigra pars compacta, along with the limbic and cingulate cortex [118]. Moreover, the striatal organization indicates varying sensitivity to BBB impairment with the striosomes having a more leaky BBB compared to the matrix [290]. This is especially interesting because Lewy bodies affect brain regions like the substantia nigra pars compacta, and several layers of the limbic cortex project predominantly to striosomes [118, 291, 292]. There are indications that dopaminergic neurons of the substantia nigra pars compacta are more prone to α-synuclein pathology due to their high levels of dopamine and its reactive metabolites [293]. Depletion of dopamine in in-vitro experiments prevented neurotoxic effects of α-syunclein observed in dopaminergic neurons [294]. In addition, the substantia nigra also has a high microglia density. Together with the elevated levels of dopamine, this constitutes a high endotoxic burden [295]. If their striatal synaptic terminals are exposed to toxins from the blood due to a leaky BBB, then this could represent a very early pathogenic event, perhaps even prior to α-synuclein pathology.

Intranigral injection of LPS into wild-type rats induces widespread microglia reactivity, decreased dopamine levels in the substantia nigra and the striatum, along with loss of tyrosine hydroxylase-positive neurons [296, 297]. Dopaminergic neurodegeneration could also be induced by intranigral administration of blood-borne molecules like histamine and thrombin [298, 299]. As dexamethasone, a potent anti-inflammatory glucocorticoid, prevents the neurodegenerative effect of LPS, it can be argued that inflammation is the primary cause for neuronal death [296]. However, it is known that dexamethasone stabilizes the BBB, which could have contributed to the preventive effects [300, 301]. Interestingly, intranigral injection of VEGF, a potent BBB disruptor, could mimic several histopathological hallmarks found in PD [301]. Interestingly, VEGF injection not only disrupts the BBB but also reversibly inhibits the P-gp transporter activity by inducing a signal pathway that removes P-gp from the plasma membrane [302, 303]. Moreover, the disruption of the BBB correlates with a loss of tyrosine hydroxylase-positive neurons in the substantia nigra, and although a strong microglial and astroglial response has been detected, the primary cause remains the breakdown of the BBB [301]. Vesicles containing oligomeric α-synuclein that circulate in the blood, are suggested to reach the brain following BBB disruption [133, 153]. In an in-vivo experiment, derived extracellular vesicles from red blood cells of patients were injected intravenously into transgenic A53 mice [304]. These extracellular vesicles containing oligomeric α-synuclein were taken up by astrocytic end-feet surrounding the BBB [304]. The study also indicated that uptake of α-synuclein-containing vesicles led to an astrocytic dysfunction. Decreased glutamate uptake was observed, likely due to an interaction between excitatory amino acid transporter 2 and the oligomeric α-synuclein [304]. High levels of glutamate in the synaptic cleft led to loss of synapses [304]. Similar pathomechanisms are suggested to be involved in PD pathogenesis [305]. α-Synuclein crossing a weakened BBB via astrocyte-mediated uptake could represent an important connection between the BBB and PD pathogenesis. Thus, an impaired BBB could induce or accelerate inflammation, α-synuclein pathology and neurodegeneration seen in PD. Therefore, stabilizing and protecting the BBB may be beneficial in early as well as in late disease stages.

On the contrary, progressive α-synuclein pathology could also be a contributing factor to BBB alterations [258, 265]. Particularly, activation of endothelial cells and their expression of cell adhesion molecules (ICAM-1, VCAM-1) could contribute to invasion of peripheral immune cells [306], further increasing inflammation.

In conclusion, current data are insufficient to state whether BBB alterations precede and cause and/or result from inflammation and α-syunclein pathology. Yet, it is certain that a deficient BBB would contribute to disease pathogenesis in a self-perpetuating cycle, and blood-borne immune cells or toxins could further accelerate neuroinflammation and Lewy body pathology. High dopamine levels, increased microglia density and age-associated BBB breakdown render affected brain regions more vulnerable to this vicious cycle. Further research is required to shed light onto BBB alterations as a pathogenic mechanism in PD.

## Potential implications of an altered BBB for therapy

If BBB alterations are part of PD pathophysiology and accelerate its progression, then protecting and strengthening the BBB may be beneficial for patients and slow down disease progression. However, to our knowledge there are currently no such strategies tested for PD.

Of note, an altered BBB will impact current and future therapeutics, as almost every substance or particle needs to cross and will thereby encounter its pathology. Small, uncharged and highly lipophilic molecules have a high influx rate across the BBB [307], but PD BBB alterations like thickened basement membrane, altered transporter function/expression and inflammatory conditions could impact the efficacy of a therapy. This could lead to decreased or increased drug penetration. Positively or negatively charged drugs interact differently with the endothelial glycocalyx and may also show different binding characteristics or transporter affinities if the glycocalyx is altered in PD [47, 49, 308]. Passive diffusion of substances into the brain parenchyma may be reduced due to the thickening of basement membrane and the decreased vascularization [119, 309]. Importantly, drugs targeting α-synuclein could be trapped in endothelial cells where the protein accumulates. This could lead to negative outcomes of clinical trials, although the treatment could have been effective. Increased penetration of drugs could in fact be detrimental. For instance, the effect of levodopa could be compromised if even small amounts of *L*-amino acid decarboxylase inhibitors (carbidopa, benserazide) could enter the brain [18]. Therapeutic strategies using these drugs are based on their inability to cross the BBB to effectively inhibit peripheral decarboxylase to enhance availability of levodopa to the brain [310]. However, benserazide can enter the brains of 6-OHDA-injected rats through the compromised BBB [311, 312]. Due to the reduced function of the efflux pump P-gp, commonly prescribed drugs such as verapamil could unexpectedly cross the BBB [141, 313]. If PD patients are prescribed drugs that rely on efflux pump or, under healthy conditions, do not cross the BBB, changes in the BBB could lead to increased exposure of the brain to these drugs. Potentially, this could lead to CNS side effects due to augmented pharmacodynamics in the brain [313]. To raise awareness to potential CNS side effects, studies using PET imaging for substances entering the brain via diffusion and also for P-gp substrates could be conducted in PD patients [313]. Animal experiments using PD models or aged animals to assess brain-uptake and resulting effects could help predict side-effects and consequences of brain entry in humans [313]. However, basement membrane thickening and degraded microvasculature could impact diffusion and distribution of drugs negatively and thereby reduce effects of P-gp reduction [313]. Regarding restoration of BBB functionality, downregulation of P-gp could be reversed using treatment with 1α,25-dihydroxyvitamin D3 as shown in a human α-synuclein PFF mouse model [146]. Alternatively, inhibiting P-gp degradation in the 26S proteasome (ubiquitin–proteasome system) could lead to higher availability of the efflux pump [314–316]. Preventing P-gp ubiquitination can be achieved in mice via inhibition of the enzyme E1 responsible for ubiquitination [315, 317].

Drugs can also reach the brain through the use of carrier- or receptor-mediated transport systems if they structurally mimic the substrates of those transporters [307]. Moreover, generating bispecific antibodies which recognize two different epitopes can be useful [318]. The bispecific antibody first binds to the carrier- or receptor-mediated transport system and, once the BBB is overcome, functions as the therapeutic drug. This has recently been used to shuttle the entire and fully functional enzymes to the brain and into subcellular compartments using the transferrin receptor: the enzyme β-glucocerebrosidase coupled to TfR-binding moieties and the amyloid-beta antibody gantenerumab fused also with the TfR-binding moieties have been successfully transferred to the brains of mice and non-human primates [319, 320]. Interestingly, utilizing specific carrier- or receptor-mediated transport systems can offer highly specific targeting of a brain region because the expression of cell- and region-specific receptors varies throughout the brain [321]. Neuropeptides or nanoparticles could target altered proteins of the BBB and thereby accumulate specifically in affected brain regions [18]. For instance, if VCAM-1 is upregulated explicitly in striatal regions as shown in Thy1-αSyn mice, then it could be used to concentrate drugs in the striatum [18, 257]. Targeting molecules like VCAM-1, ICAM-1 or αvβ3-integrin at the competitive binding site could reduce leukocyte infiltration or angiogenesis [18].

A currently heavily studied and discussed method to overcome the BBB is microbubble-enhanced focused ultrasound (FUS). FUS waves open the BBB shortly in specific sites [322]. Intravenously injected microbubbles lower the frequency, which increases the safety [322]. This method has been tested with antibodies, chemotherapy and nanoparticles [322]. Repeatedly opening of the barrier in primates does not alter behavior or brain activity observed on electroencephalography; however, slight edema was seen on MRI [323, 324]. In one study 10 participants each received 3 times of FUS BBB opening and a clinical check-up was done after 12 months [325]. During FUS administrations, BBB was opened but closed within 24–48 h [325]. There was no change in cognitive parameters in the follow-up assessment compared to controls [325]. Several other studies have demonstrated the safety of this method [322, 324, 326, 327]. However, several studies did find some negative effects including local cell apoptosis, astroglial activation and minor, transient erythrocyte leakage [326, 327]. Others reported alterations similar to a sterile infection like microglial activation, ICAM-1 upregulation and macrophage infiltration [328]. High doses of microbubbles as well as higher sonication conditions can impact the NF-кB signaling pathway and upregulate TNFα and other pro-inflammatory cytokines [329, 330]. An increase of inflammatory stimuli in brain regions associated with α-synuclein pathology should be considered carefully. While pro-inflammatory effects seem to be transient, they could still further promote pathology. Placing the FUS-induced BBB opening further away from brain regions affected by α-synuclein pathology requires a therapeutic compound that can penetrate specifically within brain tissue to eventually reach the destined brain region. Combining anti-inflammatory medication with FUS-induced BBB opening could be beneficial. This requires further studies focusing on the effects of BBB opening on α-synuclein pathology and inflammation.

Recently, transplantation of iPSC-derived pericytes has been developed [331]. Transplanted pericytes seem to promote regeneration and restoration of the BBB and thereby enhance integrity of the barrier [331]. When transplanted into a murine stroke model, iPSC-derived pericytes could successfully improve neurological function and BBB integrity [332]. While there is still no causative link between PD and pericyte degeneration or dysfunction, pericyte transplantation could stabilize the integrity of the BBB in PD [331].

To conclude, BBB alterations could influence not only pathology and progression of PD but also therapeutic interventions. Taking possible BBB impairments into account and restoring the BBB could optimize the treatments and the well-being of patients. Moreover, BBB alterations in PD patients should be considered when assessing the efficacy of new therapeutic strategies.

## Conclusion

Separating the peripheral blood circulation from the neuronal brain tissue, hence allowing the brain to be independent from ion fluctuations and protecting it against circulating toxins, emphasizes the importance of a functional BBB. In brain tissues from PD patients, altered tight junction protein levels as well as extravasation of blood molecules, like albumin, fibrinogen and erythrocytes, already indicate that the barrier function is impaired. Altered transporter protein levels and inflammation markers, accompanied by characteristics of vessel degeneration but also of angiogenesis, comprise the most important alterations found at the BBB of PD patients (Fig. 1). Inflammatory processes in PD seem to play an important role in the development and progression of BBB alterations and could thereby perpetuate the disease progression. This can be mirrored and studied in animal models (Table 1). Stabilizing the BBB may therefore represent a therapeutic and testable target. Importantly, structural and functional changes of the BBB could interfere with therapeutics. Exploiting altered structures, for example increased transporter levels or increased cell adhesion molecules, could open a novel strategy for targeting affected brain regions.

## Data Availability

The datasets used and/or analyzed during the current study are available from the corresponding author on reasonable request.

## Abbreviations

6-OHDA: 6-Hydroxydopamine
BAC: Bacterial artificial chromosome
BBB: Blood–brain barrier
CAMs: Capillary-associated microglia
CAT: Cationic amino acid transporter
CNS: Central nervous system
EAAT: Excitatory amino acid transporter
FITC: Fluorescein isothiocyanate dextran
FUS: Focused ultrasound
Glut1: Glucose transporter-1
ICAM-1: Intercellular adhesion molecule-1
ICD: Intracellular domain
IL: Interleukin
iPSCs: Induced pluripotent stem cells
LAT: L-type amino acid transporter
LPS: Lipopolysaccharide
LRP-1: Low-density lipoprotein receptor related protein-1
MCT: Monocarboxylate transporter
MMP: Matrix-metalloprotease
MPTP: 1-Methyl-4-phenyl-1,2,3,6-tetrahydropyridine
PD: Parkinson’s disease
PFF: Preformed-fibrils
P-gp: P-glycoprotein
PPARy: Proliferator activated receptor γ
pS129-αSyn: α-Synuclein phosphorylated at serine 129
RAGE: Receptor for advanced glycation end products
SNCA: α-Synuclein
TfR: Transferrin receptor
TNF: Tumor necrosis factor
UDPRS: Unified Parkinson’s disease rating scale
VCAM-1: Vascular adhesion molecule-1
VEGF: Vascular endothelial growth factor
